# Species identification of sunfish specimens (Genera *Mola* and *Masturus*, Family Molidae) from Australian and New Zealand natural history museum collections and other local sources

**DOI:** 10.1016/j.dib.2018.07.015

**Published:** 2018-07-10

**Authors:** Marianne Nyegaard, Etsuro Sawai

**Affiliations:** a School of Veterinary and Life Sciences, Murdoch University, 90 South Street, Murdoch 6150, Western Australia, Australia; b Ocean Sunfishes Information Storage Museum, C–102 Plaisir Kazui APT, 13–6 Miho, Shimizu-ku, Shizuok 424-0901, Shizuoka, Japan

## Abstract

This data-in-brief comprises a summary of sunfish specimens (Genera *Mola* and *Masturus*, Family Molidae, >29 cm total length) from natural history museum collections and other sources, such as strandings, in Australia and New Zealand. Each specimen was evaluated morphologically and identified to lowest possible taxon based on recent advances in the understanding of the *Mola* taxonomy. References to phylogenetic analyses, where applicable, are included. The summary was collated in support of publication *Giant jelly eaters on the line: species distribution and bycatch of three dominant sunfishes in the Southwest Pacific*[1].

**Specifications Table**

**Table:** 

Subject area	*Biology*
More specific subject area	*Systematics, taxonomy, morphology*
Type of data	*Tables, images*
How data was acquired	*Specimen morphology was assessed in person or from photographs. Phylogenetic information was sourced from published literature.*
Data format	*Summary incl. metadata, images*
Experimental factors	*n/a*
Experimental features	*n/a*
Data source location	Specimens are from the coastline and coastal waters of Australia and New Zealand (11°S – 168°S; 113°E – 179°E), held in museum collections in Adelaide (SAMA), Auckland (AIM), Brisbane (QM), Christchurch CMC), Dunedin (OMNZ), Hobart (TMAG), Melbourne (NMV), Perth (WAM), Sydney (AMS), Wellington (NMNZ), and London, UK (BMNH). Other material from stranding events in Australia and New Zealand is also included.
Data accessibility	Information on museum specimen held in Australian collections is available from the Online Zoological Collections of Australian Museums (OzCam) (http://ozcam.org.au). Genetic sequences are accessible from GenBank at the National Center for Biotechnology Information (NCBI) (https://www.ncbi.nlm.nih.gov) and/or the Barcode of Life Data (BOLD) System (http://www.boldsystems.org).

**Value of the data**

• Provides a comprehensive overview, including images, of sunfish specimens (genera *Mola* and *Masturus*) currently held in museum collections in Australia and New Zealand.
• Includes specimens not lodged with a museum, but for which samples are held by the authors.
• Resolves several errors in sunfish species identities of museum specimens, based on recent advances in the taxonomic understanding of the genus *Mola*.
• Provides information for researchers on where specimens and samples are held, for future research and collaboration.

## 1 Data

Table 1 lists material of ocean sunfishes (genera *Mola* and *Masturus* > 29 cm) held in Australian and New Zealand natural history museum collections. Table 2 lists specimens from Australian and New Zealand obtained from other sources, but which are not lodged with a natural history museum. The tables contain specimen detail, morphological assessment, Accession numbers for genetic sequences and references to phylogenetic studies, where applicable, and a verified or updated species identity. Table 3, Table 4 contain images, where available, of specimens in Table 1, Table 2.

**Table 1:** Whole museum specimens (>29 cm), and other *Mola* spp. and *Masturus lanceolatus* (Liénard, 1840) material identifiable to species level, held in collections in Australia and New Zealand. Museum codes according to Sabaj (2016), except ABTC (Australian Biological Tissue Collection), OMNZ (Otago Museum) and WRM (Whanganui Regional Museum). State/country abbreviations are New South Wales (NSW), New Zealand (NZ), Northern Territory (NT), South Australia (SA), Tasmania (TAS), Victoria (VIC), Western Australia (WA), Queensland (QLD). Species identity established from morphology (**MORPH**), and/or phylogeny (**PHYL**) based on mtDNA D-loop and/or Cytochrome *c* oxidase 1 (CO1) loci. Taxonomic features assessed in person (**pers**), via photographs (**photo**) and/or X-rays (**xray**): protruding snout (**PS**), head bump (**HB**), chin bump (**CB**), dorso-lateral ridge (**DLR**), ventro-lateral ridge (**VLR**), clauvs shape (**CS**), smooth band back-fold (**SBBF**), number of ossicles (**NOs**), state of paraxial ossicles (**POs**), scale morphology (**SM**). Some features could not be assessed (**n/a**).

Museum registration number and specimen details	Basis for specimen identification in this study; DNA sequence accession numbers in parentheses.	Original museum ID	Species reassignment
**AIM MA29864**, 51.1 cm TL, female, wet specimen, Poor Knights Islands, NZ (35.488°S 174.729°E^a^) 8 November 1969.	**MORPH (pers, xray)**: [6].	*M. mola*	*M. tecta*
**AIM MA30933**, 102 cm TL, cast, likely from NZ waters.	**MORPH (pers)**: [6].	*M. mola*	*M. tecta*
**AIM MA30934**, 211 cm TL, cast, likely from NZ waters.	**MORPH (pers)**: [3].	*M. mola*	*M. alexandrini*
**AMS I.2742**, 174 cm TL^b^, mounted skin, Manly Beach, NSW (33.800°S 151.283°E), purchased, November 1882.	**MORPH (photo)**: **PS** present, **HB** & **CB** present, **DLR** & **VLR** n/a, **CS** rounded, **SBBF** n/a, **NO** n/a, **POs** n/a, **SM** n/a.	*M. mola*	*M. alexandrini*
**AMS I.9412**^c^, 254 cm TL, mounted skin in poor condition, restored in 2012, Manly, NSW (33.800°S 151.283°E), beach cast, 16 December 1882 (1883 in [2]).	**MORPH (photo)**: **PS** present, **HB** & **CB** present (large), **DLR** & **VLR**: swollen, **CS** rounded, **SBBF** n/a**, NOs** n/a**, POs** n/a**, SM** n/a.	*M. mola*	*M. alexandrini*
**AMS I.18215-001**, 51.1 cm TL, wet specimen, East of Ulladulla, NSW (35.283°S 150.700°E), caught by FRV Kapala, 2 May 1973.	**MORPH (photo)**: **PS** absent, **HB** & **CB** + **DLR** & **VLR** absent (consistent with small specimen), **CS** rounded, **SBBF** absent, **NOs** >5 (large), **SM** consistent with *M. alexandrini* immature scale morphology on specimen NMNZ P.056071. **POs** n/a.	*M. ramsayi*	*M. alexandrini*
**AMS I.25630-001**, 60 cm TL, wet specimen, Crowdy Head, NSW (31.000°S 153.000°E), trawler, September 1985.	**MORPH (photo)**: **PS** absent, **HB** & **CB** + **DLR** & **VLR** absent (consistent with small specimen), **CS** rounded, **SBBF** absent, **NOs** >10 (large), **SM** consistent with *M. alexandrini*[3]. **POs** n/a.	*M. mola*	*M. alexandrini*
**AMS I.38997-001**, 250 cm TL, tissue & photographs, off Jervis Bay, NSW (35.05°S 150.733°E), caught on bow of MV “Goliath”, 13 October 1998.	**MORPH (photo)**: **PS** absent, **HB** & **CB** + **DLR** & **VLR** present, **CS** rounded, **SBBF** absent. **NOs** n/a, **POs** n/a, **SM** n/a.	*M. mola*	*M. alexandrini*
	**PHYL**: JNSW-2 in [10] (D-loop: AB439109). Also in [1], [7], [8].		
**AMS I.41536-001**, 178 cm TL, tissue & photographs, Narrabeen Beach, NSW (35.716°S 151.300°E), found alive in shallow waters in poor condition (euthanized), 11 September 2002.	**MORPH (photo)**: **PS** absent, **HB** & **CB** absent, **DLR** short**, VLR** absent, **CS** rounded with indent, **SBBF** present, **NOs** 8^d^. **POs** n/a, **SM** n/a.	*M. mola*	*M. tecta*
	**PHYL:** NNSW-1 in [10] (D-loop: AB439108). Also in [1], [7], [8]. Submission to BOLD by AMS (CO1 sequence ID: AMS174-08), phylogenetic analysis in [6].		
**AMS I.42801-001**, 175 cm TL, tissue & photographs, Sussex Inlet, NSW (35.150°S 150.600°E), beach cast, 21 August 2003.	**MORPH (photo)**: **PS** present, **HB** & **CB** present (large), **DLR** & **VLR** slightly swollen (specimen not fresh), **CS** rounded, **SBBF** very faint, **NOs** >10. **POs** n/a, **SM** n/a.	*M. ramsayi*	*M. alexandrini*
	**PHYL:** SNSW-3 in [10] (D-loop: AB439110). Also in [1], [7], [8].		
**AMS I.44396-001**, 170 cm TL, tissue & photographs, near Robe, SA (37.150°S 139.750°E), beach cast, August 2007.	**MORPH (photo)**: **CS** with extension.	*Ma. lanceolatus*	*Ma. lanceolatus*
	**PHYL:** submission to BOLD by AMS (CO1 sequence ID: AMS124-08), phylogenetic analysis in [6].		
**CMC F228**, 42 cm TL, wet specimen, Scarborough Beach, Christchurch, South Island (*ca*. 43.566°S 172.761°E), beach cast, NZ, 13 July 1964	**MORPH (photo)**: presumably *M. alexandrini* from overall morphology, but insufficient characteristics visible on photograph to verify species identity.	*M. mola*	*Mola* sp.
**CMC,** no registration number available, mounted specimen.	Not assessed (no access).	*M. mola*	–
**NMNZ P.001418**, 60.2 cm TL, male, wet specimen, off Hawke’s Bay, NZ (39.417°S 177.100°E), June 1952.	**MORPH (pers, xrays)**: [6].	*M. ramsayi*	*M. tecta*
**NMNZ P.002629**, 253.6 cm TL, cast (right side), Palliser Bay, NZ (*ca*. 41.442°S 175.008°E), 7 April 1930.	**MORPH (pers)**: [3].	*M. ramsayi*	*M. mola*
**NMNZ P.002980**, 64.6 cm TL, male, wet specimen, North Rona Bay, Eastbourne, Wellington Harbour, NZ (41.267°S 174.917°E), drag net, 30 November 1960.	**MORPH (pers)**: [6].	*M. ramsayi*	*M. tecta*
**NMNZ P.005890**, 49.9 cm TL, female, wet specimen, off Great Barrier Island, NZ (36.083°S 175.583°E), trawl 36–37 m, 25 June 1973.	**MORPH (pers, xrays)**: [6].	*M. ramsayi*	*M. tecta*
**NMNZ P.006126**, 64.8 cm TL, female, wet specimen, Oriental Bay, Wellington Harbour, NZ (41.290°S 174.793°E), beach cast, 8 Dec 1974^a^.	**MORPH (pers, xrays)**: [6].	*M. ramsayi*	*M. tecta*
**NMNZ P.006345**, 38.8 cm TL, male, wet specimen, off Te Kaha, Bay of Plenty, North Island, NZ (37.650°S 177.517°E), purse seine, 2 March 1976.	**MORPH (pers, xrays)**: [3].	*M. ramsayi*	*M. alexandrini*
**NMNZ P.009864**, 121.6 cm TL, wet specimen, North of Cape Brett, North Auckland, NZ (34.917°S 174.567°E), purse sein, 27 January 1981.	**MORPH (pers)**: **CS** with extension.	*Ma. lanceolatus*	*Ma. lanceolatus*
**NMNZ P.009887**, 38.5 cm TL, male, wet specimen, off North Cape, North Auckland, NZ (35.217°S 172.467°E), 5 February 1980.	**MORPH (pers, xrays)**: [3].	*M. ramsayi*	*M. alexandrini*
**NMNZ P.033995**, 57.2 cm TL, male, wet specimen, Bay of Plenty, Opotiki Beach, NZ (37.750°S 177.333°E), November 1996.	**MORPH (pers, xrays)**: [6].	*M. ramsayi*	*M. tecta*
**NMNZ P.034187**, 79.7 cm TL, male, wet specimen, Bay of Plenty, surf at Opotiki, NZ (37.750°S 177.333°E).	**MORPH (pers, xrays)**: [6].	*M. ramsayi*	*M. tecta*
**NMNZ P.034217**, 69.8 cm TL, male, wet specimen, Bay of Plenty, Opotiki Beach, NZ (37.733°S 177.333°E), December 1996.	**MORPH (pers, xrays)**: [6].	*M. ramsayi*	*M. tecta*
**NMNZ P.034449**, 51.5 cm TL, female, wet specimen, Southern Colville Ridge, Bay of Plenty, NZ (36.392°S 176.850°E), surface longline, 17 May 1997.	**MORPH (pers, xrays):**[3].	*M. ramsayi*	*M. alexandrini*
**NMNZ P.036964**, 45.3 cm TL, female, wet specimen, Off Mahia Peninsula, Hawke’s Bay, NZ (39.083°S 178.883°E), 13 May 1999.	**MORPH (pers, xrays)**: [3].	*M. ramsayi*	*M. alexandrini*
**NMNZ P.056054**, 212.7 cm TL, male, wet specimen, Omaha Beach, north of Tawharanui Peninsula, North Auckland, NZ (36.350°S 174.785°E), beach cast, 14 May 2013.	**MORPH (photo)**: **CS** with extension.	*Ma. lanceolatus*	*Ma. lanceolatus*
**NMNZ P.056071**, 29.3 cm TL, male, wet specimen (tissue **P.056071/TS1**), Off Raglan Harbour, Taranaki, NZ (37.728°S 174.168°E), trawl, 28 December 2012.	**MORPH (pers)**: [3].	*M. ramsayi*	*M. alexandrini*
	**PHYL:**[6] (D-loop: MF158140; CO1: MF158118). Also in [1]) (D-loop).		
**NMNZ P.057679**^e^, holotype, 101.1 cm TL, wet specimen (tissue **P.057679/TS3**), male, North Taranaki Bight, west coast of North Island, NZ (38.425°S 174.150°E), trawl, 78 m, 25 December 2015.	**MORPH (pers, xrays)**: [6].	*Mola* sp. nov.	*M. tecta*
	**PHYL**: [6] (D-loop: MF158147; CO1: MF158119). Also in [1]) (D-loop).		
**NMV 32054**, large, mounted skeleton, Port Phillip Bay, Hobsons Bay, VIC (37.850°S 144.930°E), 1879.	Not assessed (no access).	*M. mola*	–
**NMV A 18725**, 90.5 cm TL, wet specimen, Port Phillip Bay, VIC (37.8670°S 144.817°E), hook and line, 28 August 1995.	**MORPH (pers)**: [6].	*M. mola*	*M. tecta*
**NMV A 26565-001**, 78.5 cm TL, wet specimen & tissue, 13th beach near Barwon Heads, VIC (38.286°S 144.456°E), beach cast, 30 April 2009.	**MORPH (pers)**: [6].	*M. ramsayI*	*M. tecta*
	**PHYL:**[1] (D-loop: MG254032).		
**NMV A 30811-001**, *ca*. 85 cm TL, tissue & photographs, Port Augusta power station, SA (*ca*. 32.545°S 137.788°E), beach cast, 23 September 2008.	**MORPH (photo, xray): PS** absent, **HB** & **CB** absent**, VLR** absent, **DLR** short, **CS** rounded with indent, **SBBF** present, **NOs** 6, **POs** separate.	*Mola* sp.	*M. tecta*
	**PHYL**[1] (D-loop: MG254031).		
**NMV A 25071-001**, *ca*. 200 cm TL, skin sample & photographs (**NMV A 25071-002**), tissue (**NMV Z 10859**), Bunurong Marine Park (38.651°S 145.693°E), beach cast, 10 April 2003.	**MORPH (photo): PS** present (small), **HB** & **CB** present (small)**, DLR** & **VLR** swollen, **CS** n/a, **SBBF** n/a, **NOs** n/a, **POs** n/a,**SM** n/a.	*Mola* sp.	*M. mola* (Pacific clade)
	**PHYL:**[1] (D-loop: MG254033). Submission to BOLD by NMV (CO1 sequence ID: FMVIC396-08), phylogenetic analysis in [6].		
**NMV A 31759-001**, 170 cm, parts & photographs, tissue (**NMV ZZ 61327**), Lake Tyers mouth, East Gippsland, VIC (37.855°S 148.101°E), beach cast, 23 March 2017.	**MORPH (photo):** Body shape consistent with *Ma. lanceolatus*, but clavus damaged (**CS** n/a).	*Ma. lanceolatus*	*Ma. lanceolatus*
	**PHYL:**[1] (D-loop: MG254034).		
**NTM S.15520-001**, 106.5 cm TL, wet specimen & tissue, Cobourg Peninsula, NT (11.117°S 132.150°E), beach cast, 7 February 2003.	**MORPH (photo): CS** with extension.	*M. ramsayi*	*Ma. lanceolatus*
	**PHYL:**[10] (D-loop: AB439120). Also in [1].		
**OMNZ VT3248**, 242 cm TL, cast (left side) from fresh specimen, Otago Harbour, Dunedin, NZ (45.883°S 170.508°E^a^), 1961.	**MORPH (pers):**[6].	*M. ramsayi*	*M. tecta*
**OMNZ VT3249**, 78 cm TL, cast (right side) from fresh specimen, Kaka Point, Clucha District, NZ, (46.367°S 169.733°E), beach cast, 7 March 1963.	**MORPH (pers):**[6].	*M. ramsayi*	*M. tecta*
**OMNZ X2017.18**, 58 cm TL, parts & photographs, Aramoana, NZ (45.766°S 170.696°E), beach cast, 9 July 2015. Tissue sample held by MN.	**MORPH (photo): PS** absent, **HB** & **CB + DLR** & **VLR** absent (consistent with small specimen), **CS**: rounded, **SBBF** absent, **NOs** >7 (large). **SM** n/a, **POs** n/a.	*Mola* sp.	*M. alexandrini*
	**PHYL:**[6] (D-loop: MF158135; CO1: MF158129). Also in [1] (D-loop).		
**OMNZ X2017.19**, 169 cm TL, female, parts & photographs, Aramoana saltmarshes, Dunedin, NZ (45.782°S 170.711°E^a^), beach cast, 18 January 2017. Tissue sample held by MN.	**MORPH (photo)**: [6].	*Mola* sp.	*M. tecta*
	**PHYL:**[6] (D-loop: MF158136; CO1: MF158130). Also in [1] (D-loop).		
**QM I10163**, 200 cm TL, cast, Burleigh Heads, Queensland, Australia (28.083°S 153.450°E), beach cast, 18 November 1968.	**MORPH (photo): PS** present (small), **HB** & **CB** present, **DLR** &**VLR** swollen, **CS** rounded, **SBBF** absent. **NOs** n/a, **POs** n/a**, SM** n/a.	*M. mola*	*M. alexandrini*
**SAMA AMSTAC1924**, presumably wet specimen, Brown’s Bay, SA (38.048°S 140.838°E), beach cast, 19 August 1982.	Not assessed (no access).	*M. ramsayi*	–
**SAMA F243**, *ca*. 134 cm TL, cast (left side), likely from SA, 1914	**MORPH (photo)**: **PS** present, **HB** & **CB** present, **DLR** & **VLR** n/a, **CS** rounded, **SBBF** absent, **NO** n/a (features appear to have been lost during cast preparation), **POs** n/a, **SM** n/a.	*M. mola*	*M. alexandrini*
**SAMA F6046**, presumably wet specimen, Port River, Adelaide, SA (34.800°S 138. 517°E), 13 November 1982.	Not assessed (no access).	*M. ramsayi*	–
**SAMA F7542**, *ca*. 90 cm TL, wet specimen & tissue (**ABTC21528**), Spencer gulf, SA (34.780°S 138.480°E), 27 June 1994^f^.	**MORPH (photo)**: [6].	*M. ramsayi*	*M. tecta*
	**PHYL:**[6] (D-loop: MF158148). Also in [1].		
**SAMA F8085**, presumably wet specimen, Port Augusta, SA (32.500°S 137.783°E), beach cast, July 1996.	Not assessed (no access).	*Mola* sp.	–
**SAMA F9303**, presumably wet specimen, Victor Harbour, SA (*ca*. 35.549°S 138.627°E), November 1999.	Not assessed (no access).	*M. mola*	–
**SAMA F3316 – F3319**, parts, Spencer Gulf, (SA 35.717°S 137.950°E), 31 July 1965.	Not assessed (no access).	*Ma. lanceolatus*	–
**TMAG D3693**, *ca*. 200 cm TL, photographs, White Beach, Tasmania (43.120°S 147.740°E), beach cast, 2003.	**MORPH (photo): PS** present (small), **HB** & **CB** present, **DLR** & **VLR** swollen, **CS** rounded, **SBBF** absent. **NO** n/a, **POs** n/a, **SM** n/a.	*M. mola*	*M. alexandrini*
**TMAG D3885**, *ca*. 50 cm TL, frozen specimen, Randalls Bay, TAS (*ca*. 43.243 E 147.137 E), 2010.	**MORPH (photo)**: presumably *M. alexandrini* from overall morphology, but insufficient characteristics visible on photograph to verify species identity. No access to specimen.	*Mola* sp.	*Mola* sp.
**TMAG D3912**, 150 cm TL, tissue & photographs, Lindisfarne, TAS (42.850°S 147.333°E), beach cast, 12 December 2014.	**MORPH (photo)**: [6].	*Mola* sp.	*M. tecta*
	**PHYL**: [6] (D-loop: MF158149). Also in [1].		
**WAM P.33481-001**, 138 cm TL, wet specimen, Augusta, WA (34.190°S 115.100°E), beach cast, August 2010.	**MORPH (pers):** present, **HB** absent, **CB** present (small), **DLR** short, **VLR** absent, **CS** rounded, **SBBF** present (very faint), **NOs** 11-12 (large), **SM** consistent with *M. alexandrini*. **POs** n/a.	*M. ramsayi*	*M. alexandrini*
**WRM 1895.39**, >200 cm TL, mounted skin (poor condition) Napier Harbour, NZ (*ca*. 39.482°S 176.894°E), ‘captured’, May 1895.	**MORPH (photo): PS** absent, **HB** & **CB + DLR** & **VLR** absent (features likely lost during extensive preparation and repair of specimen in poor condition), **CS** rounded, **NO** >10 (large). **SM+POs** n/a.	*M. Mola*	*M. alexandrini*
**BMNH 1883.11.29.22,** 229.1 cm TL, holotype of *Orthragoriscus ramsayi*, mounted skin restored in 2016, Darling Harbour, NSW (*ca*. 33.85°S 151.20°E) beach cast, 1882.	**MORPH (pers):**[3].	*M. ramsayi*	*M. alexandrini*

a Updated since [6].

b Total Length (TL) from [2].

c Specimen recently found by AMS without a label in a storage facility; based on extensive research of old records AMS conclude this is most likely AMS I.9412, or alternatively AMS I.5312, which stranded *ca*. 1871-1874, probably in Manly Harbour (M. McGrouther, AMS, pers. comm. 2017), and was highly likely also a *M. alexandrini*[2].

d Number of ossicles (NOs) from [9].

e Included in sea surface temperature analysis in [1].

f Collection year on current specimen label is 1989, but the collection authority considers this an error (R. Foster, SAMA, pers. comm. 2017).

**Table 2:** Australian and New Zealand Molidae material obtained from strandings and other sources. State/country abbreviations are New South Wales (NSW), New Zealand (NZ) and Western Australia (WA). Species identity established from morphology (**MORPH**), and/or phylogeny (**PHYL**) based on mtDNA D-loop and/or Cytochrome *c* oxidase 1 (CO1) loci. Taxonomic features assessed in person by one or both authors (**pers**) or via photographs (**photo**).

Sample number and specimen detail	Basis for specimen identification; DNA sequence accession numbers in parentheses.	Species ID by sampler	Species ID in this study
**NZ01**, 212 cm TL, Otago Harbour, NZ (45.817°S 170.617°E), beach cast, 18 February 2015. Specimen not retained, tissue held by MN.	**MORPH (photo)**: [6].	*Mola* sp. nov	*M. tecta*
	**PHYL:**[6] (D-loop: MF158134). Also in [1].		
**NZ07**, 100 cm TL, Banks Peninsula, NZ (43.833°S 172.667°E), beach cast, 30 April 2014. Specimen not retained, tissue held by MN.	**MORPH (photo)**: [6].	*Mola* sp. nov.	*M. tecta*
	**PHYL:**[6] (D-loop: MF158137; CO1: MF158120). Also in [1] (D-loop).		
**NZ08**, 151 cm TL, Banks Peninsula, NZ (43.833°S 172.667°E), beach cast, 30 April 2014. Specimen not retained, tissue held by MN.	**MORPH (photo)**: [6].	*Mola* sp. nov.	*M. tecta*
	**PHYL:**[6] (D-loop: MF158138; CO1: MF158121). Also in [1] (D-loop).		
**NZ09**, 193 cm TL, Banks Peninsula, NZ (43.833°S 172.667°E), beach cast, 30 April 2014. Specimen not retained, tissue held by MN.	**MORPH (photo)**: [6].	*Mola* sp. nov.	*M. tecta*
	**PHYL:**[6] (D-loop: MF158139; CO1: MF158122). Also in [1] (D-loop).		
**NZ12**, 155 cm TL, female, Birdling’s Flat, Banks Peninsula, NZ (43.817°S 172.700°E), beach cast, 10 May 2014. Specimen not retained, tissue held by MN, skin sample and clavus held by ES.	**MORPH (pers)**: [6].	*Mola* sp. nov.	*M. tecta*
	**PHYL:**[6] (D-loop: MF158141; CO1: MF158123). Also in [1] (D-loop).		
**NZ14**, 170 cm TL, near Birdling’s Flat, Banks Peninsula, NZ (43.833°S 172.667°E), beach cast, 14 December 2015. Specimen not retained, tissue held by MN.	**MORPH (photo)**: [6].	*Mola* sp. nov.	*M. tecta*
	**PHYL:**[6] (D-loop: MF158142; CO1: MF158124). Also in [1] (D-loop).		
**NZ16**, 261 cm TL, Kaka Point, South Otago, South Island, NZ (46.417°S 169.783°E), beach cast, 9 October 2011. Specimen not retained, tissue held by MN.	**PHYL:**[6] (D-loop: MF158143; CO1: MF158125). Also in [1] (D-loop).	*M. mola*	*M. mola* (Pacific clade)
**NZ17**^a^, 81 cm TL, male, west of South Island, NZ (41.533°S 170.933°E), purse seine, 12 April 2014. Specimen not retained, tissue held by MN, skin sample and clavus held by ES.	**MORPH (pers)**: [6].	*Mola* sp. nov.	*M. tecta*
	**PHYL:**[6] (D-loop: MF158144; CO1: MF158126). Also in [1] (D-loop).		
**NZ18**^a^^**,**^^b^, 65 cm TL, female, east of North Island, NZ (39.783°S 178.417°E), longline, 17 May 2014. Specimen not retained, genetic sample held by MN, clavus held by ES.	**MORPH (pers)**: [6].	*Mola* sp. nov.	*M. tecta*
	**PHYL:**[6] (D-loop: MF158145 & CO1: MF158127). Also in [1] (D-loop).		
**NZ19**^a^, 69 cm TL, male, east of North Island, NZ (35.150°S 176.050°E), longline, 10 August 2014. Specimen not retained, genetic sample held by MN, clavus held by ES.	**MORPH (pers)**: [6].	*Mola* sp. nov.	*M. tecta*
	**PHYL:**[6] (D-loop: MF158146; CO1: MF158128). Also in [1] (D-loop).		
**WA41**^a^, near Bremer Canyon, WA (*ca*. 34.667°S 120.133°E), propeller strike, 22 April 2015. Specimen not retained, tissue held by MN.	**PHYL:**[1] (D-loop: MG254030).	*Mola* sp.	*M. alexandrini*
**NNZ-1b**, ca. 100 cm TL, off North NZ (ca. 30.000°S 175.000°E), longline, July 2007. Specimen not retained, tissue held by ES.	**PHYL:**[3] (Electronic Supplementary Material Table S1) (D-loop: LC271189). Also in [1].	Molidae	*M. alexandrini*

a Included in sea surface temperature analysis in [1].

b Coordinates updated since [6].

**Table 3:** Photographs of museum specimens (Table 1). No photographs were available for specimens CMC (mounted specimen, no registration number), NNZ-1b, SAMA AMSTAC1924, NMV 32054, SAMA F6046, SAMA F8085, SAMA F9303, SAMA F3316 – F3319.

**Table 4:** Photographs of specimens from other sources (Table 2).

## 2 Experimental design, materials and methods

### 2.1 Museum collections

The sunfish collections held at Auckland War Memorial Museum Tamaki Paenga Hira (AIM), Wellington Museum Te Papa Tongarewa (NMNZ), Museums Victoria in Melbourne (NMV), Otago Museum in Dunedin (OMNZ), and Western Australian Museum in Perth (WAM) were examined in person, while material from the South Australian Museum in Adelaide (SAMA), Queensland Museum in Brisbane (QM), Canterbury Museum in Christchurch (CMC), Museum and Art Gallery of the Northern Territory in Darwin (NTM), Tasmanian Museum and Art Gallery in Hobart (TMAG), Australian Museum in Sydney (AMS) and Whanganui Regional Museum (WRM) sunfish collections were examined via photographs and descriptions from examinations by museum staff or volunteers. The holotype of *Orthragoriscus ramsayi* Giglioli, 1883 [now *Mola alexandrini* (Ranzani, 1839)] held at the Natural History Museum in London (BMNH 1883.11.29.22), caught off New South Wales in 1882 [2], [3] and recently reviewed [3], was also included for completeness. Museum codes are according to [4] except OMNZ and WRM.

### 2.2 Other material

Sunfish from other sources, such as strandings, were examined in person by one or both of the authors, or by volunteers. In the latter case, the morphology was also assessed from photographs by the authors.

### 2.3 Morphological assessment

Specimen morphology was assessed against relevant and recent literature [3], [5], [6]. Each specimen was identified to the lowest possible taxon, noting that the two *Mola mola* (Linnaeus, 1758) clades (Pacific vs Atlantic) [1], [8], [9] cannot be distinguished morphologically from the currently available literature [4]. For specimens examined from photographs, as many characters as possible were assessed, and a specimen identification assigned when a subjectively satisfactory combination of clear traits was available. Some of these specimens were also included in the description of *Mola tecta* Nyegaard, Sawai, Gemmell, Gillum, Loneragan, Yamanoue, Stewart, 2017, and re-description of *Mola alexandrini* [3]. Several specimens have also been included in various phylogenetic analyses elsewhere (Table 1, Table 2).

The following taxonomic features were assessed:

• Protruding snout (**SN**) – present / absent
• Head bump (**HB**) – present / absent
• Chin bump (**CB**) – present / absent
• Dorso-lateral ridge (**DLR**) – swollen / absent
• Ventro-lateral ridge (**VLR**) – swollen / absent
• Smooth band back-fold (**SBBF**) – present / absent
• Clauvs shape (**CS**) – lobed, wawy / rounded / rounded with a small indent
• Number of ossicles (**NOs**)
• Paraxial ossicles (**POs**) – merged or separate
• Scale morphology (**SM**) – according to [3]
